# Comparison among T1-Weighted Magnetic Resonance Imaging, Modified Dixon Method, and Magnetic Resonance Spectroscopy in Measuring Bone Marrow Fat

**DOI:** 10.1155/2013/298675

**Published:** 2013-03-31

**Authors:** Wei Shen, Xiuqun Gong, Jessica Weiss, Ye Jin

**Affiliations:** New York Obesity Nutrition Research Center, St. Luke's-Roosevelt Hospital and Institute of Human Nutrition, Columbia University, 1090 Amsterdam Avenue, 14K, New York, NY 10025, USA

## Abstract

*Introduction*. An increasing number of studies are utilizing different magnetic resonance (MR) methods to quantify bone marrow fat due to its potential role in osteoporosis. Our aim is to compare the measurements of bone marrow fat among T1-weighted magnetic resonance imaging (MRI), modified Dixon method (also called fat fraction MRI (FFMRI)), and magnetic resonance spectroscopy (MRS). *Methods*. Contiguous MRI scans were acquired in 27 Caucasian postmenopausal women with a modified Dixon method (i.e., FFMRI). Bone marrow adipose tissue (BMAT) of T1-weighted MRI and bone marrow fat fraction of the L3 vertebra and femoral necks were quantified using SliceOmatic and Matlab. MRS was also acquired at the L3 vertebra. *Results*. Correlation among the three MR methods measured bone marrow fat fraction and BMAT ranges from 0.78 to 0.88 (*P* < 0.001) in the L3 vertebra. Correlation between BMAT measured by T1-weighted MRI and bone marrow fat fraction measured by modified FFMRI is 0.86 (*P* < 0.001) in femoral necks. *Conclusion*. There are good correlations among T1-weighted MRI, FFMRI, and MRS for bone marrow fat quantification. The inhomogeneous distribution of bone marrow fat, the threshold segmentation of the T1-weighted MRI, and the ambiguity of the FFMRI may partially explain the difference among the three methods.

## 1. Introduction

Recent studies revealed a negative relationship between bone marrow fat and bone mineral density [1–10]. These studies, along with the cellular level evidences [6, 11–13], suggest that bone marrow fat might play a role in the pathogenesis of osteoporosis [7, 12, 14]. 

Previous studies have used different methods to measure bone marrow fat. Among the magnetic resonance methods, there are T1-weighted magnetic resonance imaging (MRI), magnetic resonance spectroscopy (MRS), and Dixon method. Each method has its comparative strengths and weaknesses. The T1-weighted MRI is a conventional practice that is familiar to all MR technologists and is therefore not technically demanding in terms of acquisition. T1-weighted MRI also requires less acquisition time than the Dixon method. The Dixon method, also called the water-fat imaging method, fat-water imaging method, or fat fraction MRI (FFMRI), represents a category of magnetic resonance methods that generates water and fat images. So far, there is no consensus on the naming of this group of methods, and for consistency's sake we use FFMRI in the present paper. MRS methods are considered the golden standard in measuring tissue fat but require the technician to prescribe the volume of interest—MRS box in the exact desired location. Consequently, the acquisition of MRS is relatively technical demanding. 

Although T1-weighted MRI, MRS, and FFMRI methods have been compared in measuring subcutaneous adipose tissue, visceral adipose tissue, organ fat (i.e., liver), it is important to compare these methods in measuring bone marrow fat for the following reasons: fat fraction of subcutaneous and visceral adipose tissue is high (i.e, ~80%), while fat fraction for liver fat is lower (i.e., <50%); in previous results, comparisons do not cover the full range of fat fraction. In addition, fat within subcutaneous and visceral adipose tissue and liver is usually homogenously distributed. Conversely, bone marrow fat can distribute inhomogeneously, and its fat fraction can range from 0 to 80% depending on the specific imaging pixel's composition of red marrow and yellow marrow in the pixel [15]. Therefore, comparisons of different MR methods in measuring subcutaneous, visceral adipose tissue or liver fat cannot necessarily be generalized to bone marrow fat measurement. The present report compares T1-weighted MRI, MRS, and FFMRI methods for measuring marrow fat in the L3 vertebra and femoral necks in a group of postmenopausal women.

## 2. Methods

### 2.1. Protocol and Design

A total of 27 Caucasian postmenopausal women (age ≥ 50 yrs, BMI 17.4–37.9 kg/m^2^) were recruited for the present study. All subjects were established as healthy and completed a medical history screening. Subjects were excluded from undergoing MRI if they had contraindications to MRI such as metal implants, claustrophobia, or weight greater than 300 lbs as per specifications of the scanner manufacturers.

### 2.2. Magnetic Resonance Imaging

#### 2.2.1. Fat Fraction MRI

**Figure 1:**
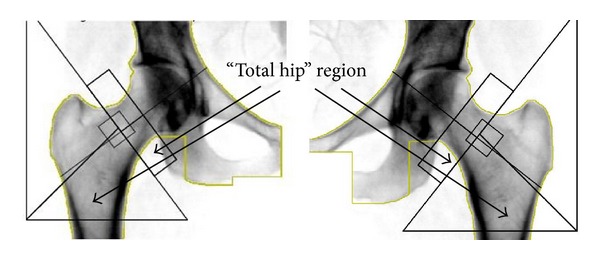
Dual-energy X-ray absorptiometry total hip scan region, which is the sum of the rectangle and triangles and is also the region we used for bone marrow fat quantification of the femoral necks in the present study.

Whole-body MRI scans were acquired, as previously reported [16], using a 1.5 T Sigma “LX” system (General Electric, Milwaukee, WI, USA). The protocol involved acquisition of 10 mm thick axial images contiguously of the whole body with a matrix of 256 × 160. Imaging is performed by using a breath-hold dual-echo spoiled gradient-recalled echo sequence (repetition time/echo time (TR/TE), 150 ms/2.1 ms, 4.4 ms) acquired with flip angles of 70° and then 20° to provide T1-weighted and intermediate-weighted images, respectively [16]. A third T1-weighted dual-echo gradient-echo breath-hold gradient-recalled echo sequence (TR/TE, 200 ms/4.6 ms, 9.2 ms; flip angle, 70°) is also performed to calculate T2*. The percentage of bone marrow fat is estimated from both sets of images, and T2* correction is applied. The dual-flip angle images are used to identify whether water or fat is the dominant constituent as previously described [16]. The images were postprocessed in Matlab (MathWorks Inc., Natick, MA, USA) to calculate fat fraction. Bone regions were manually analyzed at the Image Analysis Lab in the New York Obesity Nutrition Research Center by trained, quality-controlled, and cross-validated technicians using image analysis software (SliceOmatic, Tomovision Inc., Montreal, Canada). The bone regions for fat fraction calculation in the present study include the whole L3 vertebra and the femoral neck regions that match the “total hip” regions of the dual-energy X-ray absorptiometry scan of the subject (Figure 1). The intra- and interobserver CV for FFMRI analysis are 0.9% and 2.2%.

#### 2.2.2. T1-Weighted MRI

BMAT of the L3 and femoral necks on T1-weighted MRI (TR/TE, 150 ms/4.4 ms, flip angle, 70°) was segmented at the Image Analysis Laboratory by trained, quality-controlled, and cross-validated technicians using image analysis software (SliceOmatic, Tomovision Inc., Montreal, Canada). The threshold for BMAT segmentation on T1-weighted MRI was set at the same level as subcutaneous adipose tissue on the grey scale. The reader first sets the threshold that best segments subcutaneous adipose tissue on the grey scale [2–4, 17–19], then that threshold is used in the same image to segment BMAT. In the SliceOmatic software package, the segmentation threshold can be freely adjusted and the analyst can view the “preview” of subcutaneous adipose tissue segmentation, which is transparently overlaid on the grey image. When the “preview” of the segmentation best matched the subcutaneous adipose tissue, the corresponding threshold was determined as the threshold to segment BMAT. Tissue compartment volume was calculated as previously described [20]. The intra- and interobserver CV for T1-weighted MRI analysis are 1.0% and 2.6%.

### 2.3. Magnetic Resonance Spectroscopy

Spine phase-array coil was used for standard PRESS sequence (P.A. Bottomley, US Patent 4480 228 (1984)) MRS acquisition [21]. A PRESS box with dimensions w/2·d/2·h/2 cm^3^ was located centrally in the L3 vertebral body (TR/TE 3000/25) [21, 22]. Fat fraction was calculated after spectra are processed by jMRUI (available at http://www.mrui.uab.es/mrui/mrui_Overview.shtml) [21, 22]. Manually selected resonance frequency and line width of water (4.65 ppm) and fat (1.3 ppm) peaks were used as starting values in the nonlinear least squares fitting algorithm. Fat fraction, defined as the relative fat signal intensity amplitude in terms of a percentage of total signal intensity amplitude (*S*_fat_ and *S*_water_), was calculated according to the following equation [6]: Fat fraction = *S*_fat_/(*S*_fat_ + *S*_water_). The intra- and interobserver CV for MRS are both 0%, due to the automatic process of the algorithm.

### 2.4. Statistical Methods

Pearson correlation coefficients among bone marrow fat measurements of different methods were calculated for the L3 vertebra and femoral necks. When necessary, variable values were mathematically transformed to normalize the residual distributions. Log transformations were applied initially and followed by Box-Cox transformations if necessary [23].

All statistical analyses were carried out using SAS 9.2 package (SAS Institute. Inc., Cary, NC, USA). Two-tailed (*α* = 0.05) tests of significance were used.

## 3. Results 

### 3.1. Descriptive Statistics

All subjects (*n* = 27) were postmenopausal Caucasian women and ranged in age from 51 to 61 years (mean ± SD, 55.2 ± 3.3 years). BMI ranged from 17.8 to 37.9*** ***kg/m^2^ (mean ± SD, 24.2 ± 4.9* *kg/m^2^).

### 3.2. Relationship of Bone Marrow Fat Measurement among T1-Weighted MRI, MRS, and FFMRI in L3 Vertebra

**Figure 2:**
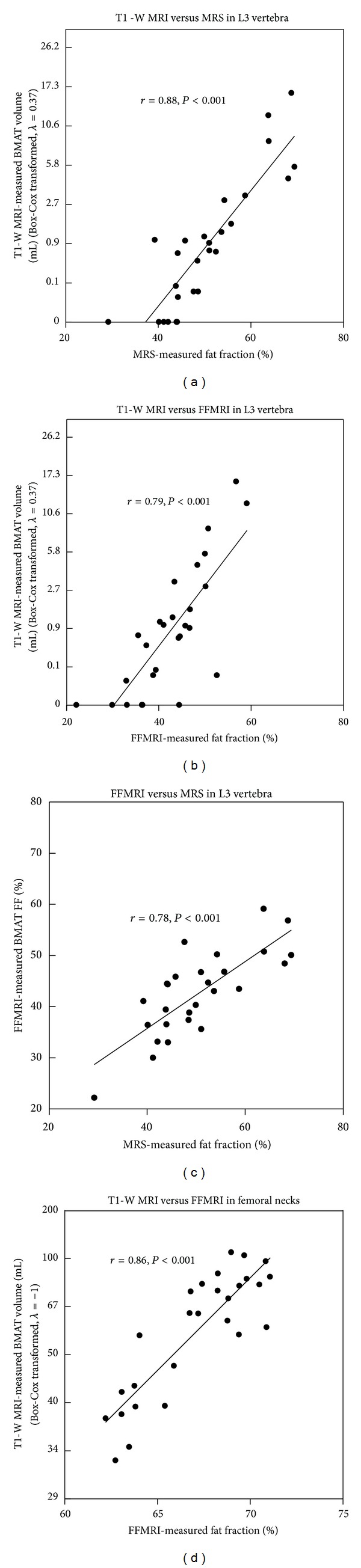
(a) Correlation between BMAT measured by T1-weighted MRI (T1-W MRI) and bone marrow fat fraction (FF) measured by MRS in L3-vertebra; (b) correlation between BMAT measured by fat fraction MRI (FFMRI) and bone marrow fat fraction measured by MRS in L3-vertebra; (c) correlation between BMAT measured by T1-weighted MRI and bone marrow FF measured by FFMRI in L3-vertebra; (d) correlation between BMAT measured by T1-weighted MRI and bone marrow FF measured by FFMRI in femoral necks.

For bone marrow fat measurement in the L3 vertebra, the correlation between Box-Cox-transformed T1-weighted MRI and MRS is 0.88 (*P* < 0.001) (Figure 2(a)). The correlation between T1-weighted MRI and FFMRI was 0.79 (*P* < 0.001) (Figure 2(b)). The correlation between MRS and FFMRI was 0.78 (*P* < 0.001) (Figure 2(c)). We further located the region on FFMRI that best matched the MRS box; the correlation between MRS and FFMRI improved to 0.86 (*P* = 0.004) (plot not shown).

### 3.3. Relationship of Bone Marrow Fat Measurement among T1-Weighted MRI and FFMRI in Femoral Necks

For bone marrow fat measurement in femoral necks, the correlation between Box-Cox-transformed T1-weighted MRI and FFMRI was 0.86 (*P* < 0.001) (Figure 2(d)). MRS was not acquired in the femoral necks.

## 4. Discussion

This study compared bone marrow fat measured by three magnetic resonance methods: T1-weighted MRI, MRS, and FFMRI. We have shown good correlations among the three methods. We chose the L3 vertebra and femoral neck because (1) these are the locations that are most frequently used to measure bone marrow fat; (2) a major interest in bone marrow fat measurement is attributed to its relationship with osteoporosis; femoral neck and lumbar spine are the locations used for the diagnosis of osteoporosis. We did not do absolute comparison among the three magnetic resonance methods because the scales of the results of these methods were not the same. In addition, T1-weighted MRI measures adipose tissue amount, while MRS and FFMRI measured fat fraction. Although we used these terms interchangeably, fat and adipose tissue are not the same components [24]. Fat makes up ~80% of adipose tissue, with the rest as water, proteins, minerals, and so forth.

The discrepancy among the three methods can be attributed to several factors. First, MRS measures ~1/8 (i.e., w/2·d/2·h/2) of the total volume of the L3 vertebra, and the MRS volume of interest is located at the center of the vertebra. On the other hand, T1-weighted MRI and FFMRI both measure the entire L3 vertebra. If the distribution of adipose tissue in the cavity of the L3 vertebra is not homogeneous, fat fraction of MRS may not reflect that of the entire vertebra [15]. When we calculated fat fraction on FFMRI in the region that best matches the MRS box region, the correlation between FFMRI and MRS improved (i.e., *r* = 0.86 versus 0.78, *P* = 0.004). However, because MRI was acquired at 1 cm slice thickness, and the L3 vertebra had a height of 2.4–2.9 cm (measured at the center of the vertebra) in this study, FFMRI was subjected to partial volume effect. Another error source of FFMRI was due to the miscalculations at approximately 45% fat content. The FFMRI method use of in-phase and out-of-phase gradient-echo MR imaging was performed with dual-flip angles (70°, 20°) to resolve ambiguity of the dominant constituent (i.e., water or fat). There were algorithmic miscalculations at approximately 45% fat content because of crossover of estimated fat curves [16]. Therefore, pixels of approximately 45% fat content could have been influenced. It should be noted that there are many versions of FFMRI methods available and error source of these methods may be different from the FFMRI method used in the present study both qualitatively and quantitatively [25–27].

The error source of T1-weighted MRI can be attributed both to the partial volume effect of MRI and to the single threshold T1-weighted MRI method being semiquantitative. Only image pixels containing bone marrow adipose tissue that reach a certain threshold were quantified as BMAT on T1-weighted MRI. The T1-weighted MRI method that was used in the present study has not only been validated for quantifying regional adipose tissue volume [28, 29] but has also been widely applied to adipose tissue measurement and serves as a reference method for adipose tissue quantification [30–36]. However, bone marrow fat pixels below the threshold for subcutaneous adipose tissue were not quantified as BMAT in the present study.

### 4.1. Limitations and Future Directions

FFMRI is a fast evolving field, and there are newer water-fat imaging methods available now [25–27]. The present study only tested one version of the FFMRI methods and the limitation of this version may not necessarily apply to other FFMRI methods. The advantage of this method is that it only uses sequences that are commercially available on almost all MRI scanners. Therefore, this method may be used in multicenter, large clinical trials. On the other hand, most-recently-developed FFMRI methods that are only available on certain MRI scanners may be used for smaller-scale studies that require high accuracy. Future studies may use more advanced FFMRI methods to quantify BMAT and to compare with MRS in quantifying bone marrow fat. Future studies may also evaluate how repositioning of the subject would influence the agreement of bone marrow fat quantification by different methods.

## 5. Conclusions

There is a good correlation among bone marrow fat measured by T1-weighted MRI, FFMRI, and MRS. The inhomogeneous distribution of bone marrow fat, the threshold segmentation of the T1-weighted MRI, and the ambiguity of FFMRI may partially explain the difference among the three methods in measuring BMAT.
